# Investigation of the NOTCH3 and TNFSF7 Genes on C19p13 as Candidates for Migraine

**DOI:** 10.2174/1874205X00802010001

**Published:** 2008-04-23

**Authors:** Robert A Smith, Robert Curtain, Mick Ovcaric, Lotti Tajouri, John MacMillan, Lyn Griffiths

**Affiliations:** 1 Genomics Research Centre, School of Medical Science, Griffith University, Gold Coast, Queensland, Australia; 2 Queensland Clinical Genetics Service, Royal Children’s Hospital Health Service District, Brisbane, Queensland, Australia

**Keywords:** Typical migraine, NOTCH3, CADASIL, TNFSF7, C19p13, MO

## Abstract

To investigate the migraine locus around the C19p13 region through analysis of the NOTCH3 gene (C19p13.2-p13.1), previously shown to be a gene involved in CADASIL and the TNFSF7 gene (C19p13), homologous to the ligands of TNF-alpha and TNF-beta, genes that have previously been associated with migraine. The NOTCH3 gene was analysed by sequencing all exons with known CADASIL mutations in a typical (non-familial hemiplegic) migraine family (MF1) that has previously been shown to be linked to C19p13. The TNFSF7 gene was investigated through SNP association analysis using a matched case-control migraine population. NOTCH3 gene sequencing results for affected members of MF1 proved to be negative for all known sequence variants giving rise to mutations for CADASIL. TNFSF7 gene chi-square results showed non-significant *P* values across all populations tested against controls, except for the MO subgroup which displayed a possible association with the TNFSF7 SNP (genotype, allele analysis *P* = 0.036, *P* = 0.017 respectively). Our results suggest that common migraine is not caused by any known CADASIL mutations in the NOTCH3 gene of interest. However, the TNFSF7 gene displayed signs of involvement in a MO affected population and indicates that further independent studies of this marker are warranted.

## INTRODUCTION

Typical migraine is a common debilitating disorder affecting approximately 12% of the Western population [1]. A large US epidemiological study indicated that the disease affects around 18% of women and 12 % of men in the overall population [2]. The disease has two main subtypes, migraine without aura (MO) affecting ~ 73% and migraine with aura (MA) affecting about 33%. Migraine with aura is defined as the more severe type and both subtypes can occur within the same family. The number of genes involved in common migraine is unknown; although genes for a rare severe sub-type of migraine Familial Hemplegic Migraine (FHM) have been identified on chromosome 19 [3] and recently FHM type 2 has been identified on chromosome 1 [4].

Mutations in the neuronal calcium channel gene (CACNA1A) on 19p13.1-13.2 have been shown to cause FHM [3]. Calcium channel genes may also be involved in typical migraine (MA and MO) as some studies have indicated that the FHM locus may contribute to migraine with and without aura [5]. We have previously reported linkage in one large typical migraine family [6] to the CACNA1A region on chr19. Also in a study by Terwindt *et al.* (1998) [7] the authors detected an FHM mutation in the CACNA1A gene in a typical migraine patient with MA, suggesting that FHM may be a rare and severe form of MA.

Typical migraine has been extensively studied on chromosome 19 showing positive linkage to causal markers in the C19p13 area [5,6,8-10], but only in FHM has the chromosome 19 gene and mutations within been identified [3]. The NOTCH3 gene on C19p13.2-p13.1 has previously been shown to be a gene involved in CADASIL (cerebral autosomal dominant arteriopathy with subcortical infarcts and leukoencephalopathy) [11]. CADASIL is an inherited stroke syndrome that leads to dementia. The key features of the disorder are migraine, recurrent subcortical events, and dementia, in association with diffuse white matter abnormalities on neuroimaging [12]. The gene encodes a large transmembrane receptor [13], which has been shown to be expressed in vascular smooth muscle cells in adult human tissues [14]. In a study by Joutel *et al.* 1997 [13] this large gene, containing 33 exons, was found to contain a cluster of mutations in exons 3 and 4 affecting 64% (32/50) of tested patients with the CADASIL disorder. Of the other 36% of CADASIL sufferers, 26% (13/50) have mutations in exons 2,5,11,14,18,19,22 or 23 and the other 10% (5/50) had no detectable mutation in the NOTCH3 gene [13]. Mutations identified to date in NOTCH3**have all been missense mutations involving a loss or gain of a cysteine amino acid residue [13].

The NOTCH3 gene may also be implicated in migraine, as migraine shows some symptomatic overlap with CADASIL. This has been suggested from studies performed by Hutchinson *et al. *(1995) [15] and Verin *et al. *(1995) [16] where the authors have found some correlations between the two disorders. Hutchinson *et al. *(1995) [15] used MRI to study 15 members of an Irish family, 10 of whom had evidence of CADASIL. Five members of this family had hemiplegic migraine. They proposed that hemiplegic migraine may be an allelic disorder to CADASIL. Migraine with aura (MA) is also included as one of the clinical hallmarks of CADASIL, with many CADASIL patients showing an unusually high frequency of attacks of migraine with atypical aura [17]. In 2003, Oberstein *et al. *[18] found that among 6 individuals who carried a mutation in the NOTCH3 gene, there was an increase in white matter hyperintensities on brain MRI, compared to controls and migraine with aura was more common in these subjects than in their controls. In addition, a recent association study by Schwaag *et al. *identified a significant association of the synonymous rs1043994 polymorphism with migraine [19]. However, not all NOTCH3 mutations may be involved in migraine, as an association study by Borroni *et al. *[20] indicated that the that functional polymorphism T6746C of NOTCH3 did not have any association with migraine in the tested Italian population.

The TNFSF7 gene (Tumor Necrosis Factor Ligand Superfamily, member 7) localized on C19p13 is a surface antigen found on activated, but not resting, T and B lymphocytes [21]. It is a 19 amino acid protein containing a 20-amino acid hydrophilic N-terminal domain that lacks a signal sequence; an 18-amino acid hydrophobic region that presumably functions as a transmembrane anchor; and a C-terminal domain that contains 2 potential N-linked glycosylation sites is extracellular classifying TNFSF7 as a type II transmembrane protein. TNFSF7 is homologous to the ligands of the TNF receptor family, including TNF-alpha, TNF-beta and the CD40 ligand, showing 19 to 24% amino acid sequence identity in the extracellular region. [21]. TNF-alpha and TNF-beta have both been previously associated with migraine. Rainero *et al.* 2004 [22], recently found an association between the tumor necrosis factor-alpha -308 G/A gene polymorphism and migraine. In a group of 299 migraine patients and 306 control subjects, the association of this polymorphism with the occurrence and clinical characteristics of migraine was tested. They found that homozygosity for the G allele was associated with an increased risk of migraine (odds ratio [OR] = 2.85, p <0.001) [22]. Also Empl *et al.* 2003 [23] suggested that TNF-alpha could contribute to migraine pain generation and in their study of TNF-alpha and its soluble receptor sTNF-RI, they found that migraine patients tended to have less concentration levels of sTNF-RI (794 +/- 158 pg/ml) than controls (945 +/- 137 pg/ml) [23]. The authors suggested that if TNF-alpha plays a role in migraine physiopathology, migraine patients may lack sufficient antagonistic sTNF-RI to neutralize hyperalgesic TNF-alpha during a migraine attack, causing pain [23]. The TNF-beta gene has been associated with migraine without aura. Trabace *et al.* 2002 [24] found the frequency of the TNFB*2 allele of the TNF-beta gene was significantly increased in patients with migraine without aura as compared with the control group (78.72% versus 61.4%, Pc =.004) [24]. They stated that carriage of the TNFB*2 allele confers a high risk for the development of migraine without aura. The data supported the hypothesis that TNF-beta could be a susceptibility gene in migraine without aura [24]. Since TNFSF7 is homologous to the ligands of the TNF receptor family i.e. TNF-alpha and TNF-beta, (and is localized to a migraine susceptibility area at C19p13) we decided to investigate this gene for association with migraine.

This study investigated the migraine susceptibility locus C19p13 by testing two genes, from this genomic region, that may possibly be involved in the disorder. The NOTCH3 gene was analysed by sequencing all exons with known CADASIL mutations through a family previously linked to C19p13 [6]. The TNFSF7 gene was also investigated using SNP case-control association analysis in a population of migraineurs and matched controls.

## MATERIALS AND METHODOLOGY

### Subjects

The subjects who participated in this study were all of Australian Caucasian origin and were diagnosed for migraine by a detailed questionnaire and a clinical neurologist in accordance to the International Headache Society guidelines [25]. The control population consisted of individuals who indicated on a questionnaire that they had never suffered from migraine or any similar condition and that none of their first or second degree relatives suffered from migraine or similar conditions. This research was approved by the Griffith University Ethics Committee and all subjects participating in the study gave consent. Blood samples for the pedigree population as well as the association population were collected through the Genomics Research Centre clinic. Genomic DNA, from these patients, was extracted utilizing DNA extraction methods described previously [26, 27].

**Fig. (1):**
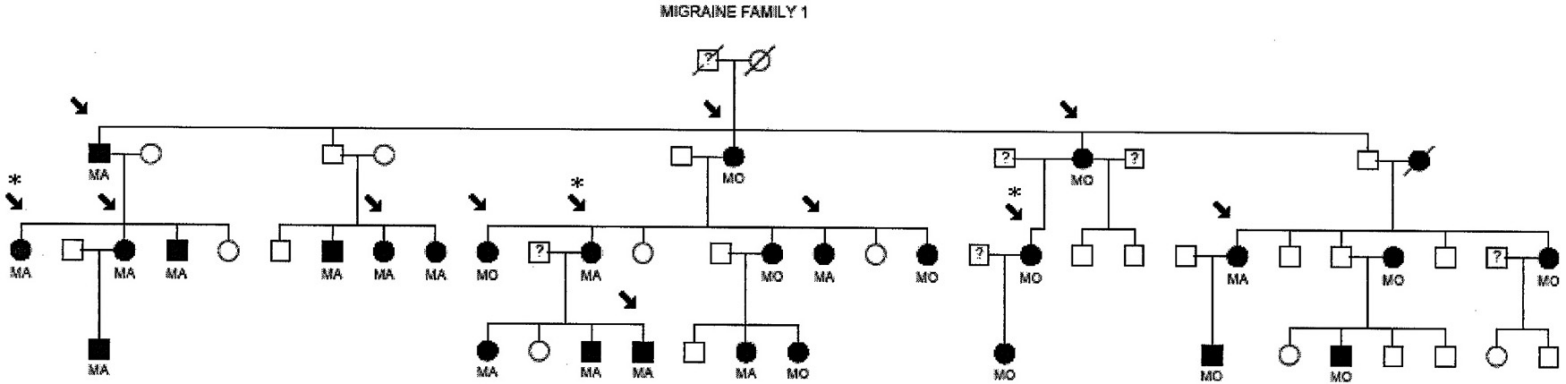
1 (MF1) Individuals in black have been diagnosed as MA or MO. Non-migraine individuals are in clear. Samples tested are indicated by an arrow. Samples  indicated by asteric (*) were sequenced for all known CADASIL mutations in the NOTCH3 gene.

For mutation analysis, 12 affected DNA samples obtained from migraine family 1 (MF1) (Fig. **1**), a pedigree showing linkage to 19p13 in a previous study [6], were screened for the known exon 3 and exon 4 CADASIL mutations (Table **1**) in the NOTCH3 gene. The pedigree family members tested (indicated by an arrow in Fig. **1**) were migraine sufferers of which eight were diagnosed MA and four MO affected, two were males (both MA affected) and ten females (six MA and four MO affected). The age of these migraine affected pedigree members range from 31 to 87 years.

Secondly 3 of the 12 family members, from three separate branches of MF1 (Fig. **1**), were further screened by sequencing the rest of all known exons containing CADASIL mutations in the NOTCH3 gene (Table **1**). The association study included a case-control population of 220 migraineurs and 220 sex, age and ethnicity matched controls.

### Mutation Analysis

Mutation screening was performed by direct sequencing of exons, exhibiting known CADASIL mutations, in the NOTCH3 gene utilizing genomic DNA samples from migraine affected pedigree members of MF1 (Fig. **1**). Templates of PCR DNA fragments were generated in the forward and reverse direction using standard PCR (outlined below in association study) and sequencing conditions (ABI protocols). Table **2** shows a list of exon primer sequences used in sequencing for the known mutations. The resulting products were electrophoresed on an ABI377 Sequencer (Applied Biosystems) and analysed using Sequencher software (Gene Codes Corporation) against a control sequence (Accession number: NM 000435).

### Association Analysis

The TNFSF7 SNP, for association analysis, was selected utilizing the software SNPbrowser^TM^ (ABI) and Ensembl Genome Browser, NCBI SNP databases. This synonymous coding SNP (refSNP 1862511, C/T variation, codes for amino acid Cysteine) is contained within exon 3 of the TNFSF7 gene with a minor allele frequency of approximately 0.3 (T allele). Primer sequences for the SNP were designed by utilizing Primer Express^TM^ v 2.0 software (ABI) and are displayed below.

Forward primer: 5’- AGCACTGGGCCGCTCC – 3’

Reverse primer: 

5’- CAAAAGTGTCCCAGTGAGGTTG – 3’

The PCR reaction utilized final concentrations of 1.75mM, 0.2µM, 200µM, 1X and 1U for MgCl_2, _Primers, dNTPs, 10x Buffer and *Taq* polymerase, respectively. Approximately 40ng of DNA per reaction was used in the PCR. Cycling conditions for PCR consisted of 94°C for 4 minutes, then 35 cycles of 94°C for 1 minute and 60°C for 1 minute. The final PCR extension consisted of 72°C for 2 minutes.

SNP genotype analysis was performed using a restriction enzyme site that overlapped the SNP site (***C***TCCTC). Restriction enzyme digest conditions utilized 10ul of PCR product and a 10ul mix of 3U of restriction enzyme with 1X enzyme buffer and H2O. Digest products were loaded and electrophoresised on 3% standard agarose gels running at 110 volts for 1 hour. The genotyping allele set and corresponding restriction digest fragment size consisted of: C allele = 192,100bp, T allele = 292bp, C/T = 292,192,100bp.

Genotype and allele frequencies for the SNP variant were calculated from observed genotype counts. The expected genotype proportions according to the Hardy-Weinberg law were calculated and compared to observed genotypes as a control for systematic genotyping error and population stratification. Genotype and allele frequencies were initially assessed for association with migraine, then MA and MO populations were investigated using conventional contingency table analyses incorporating the standard chi-squared test for independence.

## RESULTS

### Mutation Analysis

The DNA samples from MF1 that were examined for NOTCH3 – CADASIL mutations were from individuals affected with typical migraine in a pedigree previously shown to be linked to chromosome 19p13 [6]. Firstly 12 members from MF1 were screened for exon 3 and 4 mutations of the NOTCH3 gene using direct sequencing methods - a diagnostic screening process that is utilized first in detecting CADASIL mutations. The results revealed no variations or mutations detected in the sequence of the two exons within the twelve migraine family one DNA templates. Secondly, 3 members of MF1 were chosen from three separate pedigree branches to test for the rest of the exons with known CADASIL mutations in NOTCH3 (Table **1**). These results also proved to be negative with no known sequence variants detected in the extended exon sequence analysis. The sequencing results displayed that of a normal coding sequence for the NOTCH3 gene (Accession number: NM 000435).

### Association Analysis

Total distribution of the rs1862511 SNP genotype and allele frequencies of the TNFSF7 gene in Migraine (Total), MA, MO and Control Groups are displayed in Table **3**.

The results of chi-square analysis of the TNFSF7 SNP (rs1862511) are displayed in Table **4**. All population data of observed genotypes fitted the expected genotype proportions according to the Hardy-Weinberg law. Chi-square results showed non-significant *P* values (*P* > 0.05) across all populations tested against controls. The only exception was for the MO subgroup which displayed a positive association for both alleles (*P* = 0.017) and genotypes (*P* = 0.036) for the TNFSF7 SNP (Table **4**).

## DISCUSSION

The procedure for genetic diagnostic testing for CADASIL mutations has recently been revised. We undertook a similar approach to this revision in testing for these mutations in common migraine. A study in Britain [28], found 15 different point mutations in the NOTCH3 gene in 48 families, 73% of which were in exon 4, 8% in exon 3, and 6% in each of exons 5 and 6. The authors suggested that on the basis of this spectrum the suggested protocol for genetic diagnostic testing for CADASIL would be to screen exon 4 and proceed to mutational screening of exons 3, 5, and 6 where indicated [28]. A similar approach can be undertaken in diagnostic testing for other diseases with known mutations in genes causing a disorder, including FHM whereby two genes and corresponding mutations have been identified in causing this rare subtype of migraine.

The NOTCH3 gene has been localised to C19p13, a region showing linkage to MF1 a typical (not FHM) migraine pedigree. Our sequencing results for this gene, specifically testing exons with known CADASIL mutations proved to be negative. These results indicate that common migraine, at least in this pedigree, is not caused by mutations in the NOTCH3 gene.

TNF-alpha and TNF-beta have both been previously associated with migraine [22, 24]. Since TNFSF7 is localized to the same migraine susceptibility area at C19p13 and shows homology to the ligands of the TNF-alpha and TNF-beta genes (both localized at C6p21.3), we decided to also investigate this gene for involvement in migraine. A suitable informative SNP (rs1862511) was selected for an association analysis study involving a matched case-control population. Overall chi-square results comparing migraine (total) and MA sub-populations with sex, age and ethnicity matched controls proved to be non-significant, with *P* values of *P* = 0.136, *P* = 0.589 respectively obtained (Table **4**). These values were both above the threshold of *P*<0.05 for significance. However, the MO subgroup displayed signs of possible involvement of the TNFSF7 SNP with this sub-population compared to controls. *P* values for genotype and allele analysis were *P* = 0.036 and *P* = 0.017, respectively (Table **4**). These results indicate that this genetic variant may play a role in migraine without aura and warrant further investigation.

Overall the results presented here do not support a relationship between NOTCH3 and common migraine. Although CADASIL patients often display migraine symptoms there was no evidence that CADASIL mutations from the NOTCH3 gene are involved in typical migraine in our C19p13 linked [6] migraine family samples. However, this does not discount the possibility of a role for mutations in the as yet unscreened exons in NOTCH3 in migraine susceptibility. The TNFSF7 gene also localized to the C19p13 region did not show any association with typical migraine or the MA subtype, however a weak association was found with MO affected individuals. Since this gene shows homology to the TNF receptor family and both TNF-alpha and beta have previously shown association with typical migraine [22, 24], the TNFSF7 gene (localized****on C19p13, a migraine linked region, see [5,6,8-10]) was investigated in migraine. Our association study showed signs of possible involvement of this gene with the MO subtype only, but clearly more work needs to be done in increasing sample size numbers and also confirmation from other research groups to give an indication of a relationship of this gene to migraine without aura.

## CONCLUSION

It is clear that typical migraine is a complex disorder that may involve several genes on various chromosomes. Since the C19p13 region has been implicated in a number of studies it is considered a ‘hotspot’ for the disorder. More extensive sequencing of the NOTCH 3 gene that may identify novel mutations that relate to migraine should be undertaken. Further studies need to be performed with TNFSF7 and MO affected individuals and also other possible TNF receptor family homologues on C19p13, such as TNFSF9 (C19p13.3) and TNFSF14 (C19p13.3) should be tested for migraine involvement. It may be useful to explore the TNF receptor-migraine interaction further by studying these genes localized to the C19p13 region.

## Figures and Tables

**Table 1.** A List of all Published Mutations of CADASIL from the Notch3 Gene (Human Gene Mutation Data Base Cardiff

Accession Number	Codon	Exon	Nucleotide	Amino Acid
CM971054	49	2	TGT-TAT	Cys-Tyr
CM003012	54	2	tCGT-TGT	Arg-Cys
CM961042	71	3	TGGg-TGT	Trp-Cys
CM023649	76	3	gTGT-CGT	Cys-Arg
CM971055	90	3	cCGT-TGT	Arg-Cys
CM001263	93	3	TGC-TTC	Cys-Phe
CM023650	93	3	TGC-TAC	Cys-Tyr
CM971056	110	3	cCGT-TGT	Arg-Cys
CM001264	117	4	TGC-TTC	Cys-Phe
CM001265	123	4	TGC-TTC	Cys-Phe
CM003013	123	4	TGC-TAC	Cys-Tyr
CM023651	128	4	TGT-TAT	Cys-Tyr
CM971057	133	4	cCGC-TGC	Arg-Cys
CM022434	134	4	TGCt-TGG	Cys-Trp
CM014589	134	4	TGC-TAC	Cys-Tyr
CM971058	141	4	aCGC-TGC	Arg-Cys
CM023652	142	4	TTC-TGC	Phe-Cys
CM003947	144	4	TGC-TTC	Cys-Phe
CM001266	144	4	TGC-TCC	Cys-Ser
CM001267	144	4	TGC-TAC	Cys-Tyr
CM971059	146	4	cTGC-CGC	Cys-Arg
CM001268	150	4	TAC-TGC	Tyr-Cys
CM971060	153	4	cCGC-TGC	Arg-Cys
CM003014	162	4	gTGC-AGC	Cys-Ser
CM961043	169	4	cCGC-TGC	Arg-Cys
CM971061	171	4	tGGT-TGT	Gly-Cys
CM033795	174	4	cTGC-CGC	Cys-Arg
CM014211	174	4	TGC-TTC	Cys-Phe
CM001269	174	4	TGC-TAC	Cys-Tyr
CM003015	180	4	TCC-TGC	Ser-Cys
CM961044	182	4	cCGC-TGC	Arg-Cys
CM001270	183	4	cTGC-CGC	Cys-Arg
CM001271	183	4	cTGC-AGC	Cys-Ser
CM971062	185	4	gTGT-CGT	Cys-Arg
CM014590	185	4	gTGT-GGT	Cys-Gly
CM023653	194	4	aTGT-CGT	Cys-Arg
CM001272	194	4	TGT-TTT	Cys-Phe
CM003016	194	4	TGT-TAT	Cys-Tyr
CM003017	206	4	TGC-TAC	Cys-Tyr
CM003018	207	4	cCGT-TGT	Arg-Cys
CM971063	212	4	cTGC-AGC	Cys-Ser
CM033796	213	4	AGG-AAG	Arg-Lys
CM971064	222	4	cTGT-GGT	Cys-Gly
CM023654	222	4	TGT-TAT	Cys-Tyr
CM971065	224	4	TGT-TAT	Cys-Tyr
CM023655	233	5	tTGT-AGT	Cys-Ser
CM023656	251	5	aTGC-CGC	Cys-Arg
CM971066	258	5	TAT-TGT	Tyr-Cys
CM014070	332	6	cCGC-TGC	Arg-Cys
CM023657	420	8	cGGT-TGT	Gly-Cys
CM014591	428	8	TGT-TCT	Cys-Ser
CM023658	440	8	cTGC-GGC	Cys-Gly
CM023659	449	8	cCGC-TGC	Arg-Cys
CM021648	455	8	cTGT-CGT	Cys-Arg
CM961045	542	11	TGT-TAT	Cys-Tyr
CM994179	544	11	tCGC-TGC	Arg-Cys
CM961046	558	11	tCGC-TGC	Arg-Cys
CM961047	578	11	aCGC-TGC	Arg-Cys
CM003019	607	11	cCGC-TGC	Arg-Cys
CM971067	728	14	cCGC-TGC	Arg-Cys
CM023660	953	18	cGGC-TGC	Gly-Cys
CM003020	984	18	TTC-TGC	Phe-Cys
CM971068	985	18	cCGC-TGC	Arg-Cys
CM971069	1006	19	cCGC-TGC	Arg-Cys
CM994180	1015	19	cTGC-CGC	Cys-Arg
CM023661	1021	19	TAT-TGT	Tyr-Cys
CM971070	1031	19	aCGC-TGC	Arg-Cys
CM014592	1058	20	gGGT-TGT	Gly-Cys
CM971071	1231	22	cCGT-TGT	Arg-Cys
CM961048	1261	23	gTGC-CGC	Cys-Arg

www.uwcm.ac.uk/uwcm/mg/ns/1/361163.html).

**Table 2.** Primer Sequences Utilized for Sequencing of the NOTCH3 for the CADASIL Mutations

NOTCH3 region	Sequence 5'->3'
Exon 2	F-TCCTCCACCTTCCTTCAC R-ACACACAGGGCCCACTGGT
Exon 3	F-TGTGCTGCCCAACCAAGCCA R-ACTGACCACACCCCCGACTA
Exon 4	F-TAGTCGGGGGTGTGGTCAGT R-CCTCTGACTCTCCTGAGTAG
Exon 5	F-TGACCATCCTTGCCCCCTT R-CTGGCCTGTGGCACACAGAT
Exon 6	F-TGGACTGCTGCATCTGTGTG R-ACACGCCTGTGGCACAGTCA
Exon 8	F-ATCGCACTCCATCCGGCA R–ACCCACCTGCCATACAGA
Exon 11A	F-ATTGGTCCGAGGCCTCACTT R–ACCTGGCTCTCGCAGCGTGT
Exon 11B	F-CCATTCCCAACCCCTCTGTG R–TGCCTGTGCTCCTGGCTACA
Exon 14	F-TCCCTGGCCTGACTACCTTC R–CTGCAGAGGGAAGGTGAGGT
Exon 18	F-GATCCTCCCTCCCACTCCTT R–AGGTCCCCAGTAACTCCA
Exon 19	F-ACTGACTCTAAGTGCTTCCC R–AGCAGGAGGTACGTGCATGA
Exon 20	F-TGTTCCTGTGCCACTCTCCT R–ACCTCCTCTTCCCTCTCCT
Exon 22A	F-TTCCTCTTGACCACCCCTCG R–TGGCAGGCACCTGAGCGACA
Exon 22B	F-CAGGATACACTGGTTTGCGC R–TGCCACGTTATGGATCAGCC
Exon 23	F-GATCTACATGCTCCCGCTCG R–TACTCCTCCTCCATAGGCCG

**Table 3.** Total Distribution for rs1862511 SNP Genotype and Allele Frequencies of the TNFSF7 Gene in Migraine (Total), MA, MO and Control Groups

	SNP Genotypes		
Group	C/C	C/T	T/T
Total Migraine	116(53%)	88(40%)	15(7%)
MA	65(49%)	59(44%)	10(7%)
MO	51(60%)	29(34%)	5(6%)
Control	73(43%)	81(48%)	16(9%)
	SNP Alleles		
Group	C	T	
Total Migraine	320(73%)	118(27%)	
MA	189(71%)	79(29%)	
MO	131(77%)	39(23%)	
Control	227(67%)	113(33%)	

**Table 4.** Chi-Squared (χ^2^) Analysis of all Migraine Groups Against Controls for rs1862511 SNP of the TNFSF7 Gene

	Frequency Comparison	
Group	Genotypes	Alleles
Total migraine	^ ^χ^2^ = 4.00,*P* = 0.136	χ^2^ = 3.63, *P* = 0.057
MA	χ^2^ = 1.06, *P* = 0.589	χ^2^ = 0.98, *P* = 0.322
MO	χ^2^ = 6.65, *P* = 0.036	χ^2^ = 5.74, *P* = 0.017
